# The Roles of Extracellular Vesicles and Organoid Models in Female Reproductive Physiology

**DOI:** 10.3390/ijms23063186

**Published:** 2022-03-16

**Authors:** Riley E. Thompson, Gerrit J. Bouma, Fiona K. Hollinshead

**Affiliations:** College of Veterinary Medicine and Biomedical Sciences, Colorado State University, 1601 Campus Delivery, Fort Collins, CO 80523, USA; riley.thompson2@colostate.edu (R.E.T.); gerrit.bouma@colostate.edu (G.J.B.)

**Keywords:** extracellular vesicle, exosome, microvesicle, organoid, 3D culture, in vitro, reproduction, oviduct, endometrium, ovary

## Abstract

Culture model systems that can recapitulate the anatomy and physiology of reproductive organs, such as three-dimensional (3D) organoid culture systems, limit the cost and welfare concerns associated with a research animal colony and provide alternative approaches to study specific processes in humans and animals. These 3D models facilitate a greater understanding of the physiological role of individual cell types and their interactions than can be accomplished with traditional monolayer culture systems. Furthermore, 3D culture systems allow for the examination of specific cellular, molecular, or hormonal interactions, without confounding factors that occur with in vivo models, and provide a powerful approach to study physiological and pathological reproductive conditions. The goal of this paper is to review and compare organoid culture systems to other in vitro cell culture models, currently used to study female reproductive physiology, with an emphasis on the role of extracellular vesicle interactions. The critical role of extracellular vesicles for intercellular communication in physiological processes, including reproduction, has been well documented, and an overview of the roles of extracellular vesicles in organoid systems will be provided. Finally, we will propose future directions for understanding the role of extracellular vesicles in normal and pathological conditions of reproductive organs, utilizing 3D organoid culture systems.

## 1. Introduction

Reproductive physiology involves extensive communication between different organs and tissues and between different cells within tissues. A variety of models have been developed to study cellular communication in reproductive physiology. However, developing physiologically relevant in vitro models has been challenging because these dynamic models require specific cell targeting with a functional response to stimuli in a controlled environment. Therefore, the development of in vitro models that accurately mimic in vivo form and function are necessary (i) to reduce the use of live animal models and their associated financial and welfare costs, (ii) to study complex cellular processes that are difficult to isolate and study in vivo, and (iii) to avoid ethical barriers (e.g., research on human patients). Several tissue and cell culture models have been described to study reproductive physiology and pathology, including the study of complex intercellular communication between cells and tissues, involving extracellular vesicles (EVs).

Cells in vivo and in cell culture systems communicate extensively using EVs, which are discrete, lipid-bound nanoparticles that carry biological materials, including proteins and nucleic acids. Organoids are an ideal model system to evaluate the EV role in an isolated population of cells because organoids can proliferate long term, while maintaining in vivo-like physiological structure and function. Furthermore, organoids facilitate the evaluation of specific cell types without confounding interactions from other cell types and their secreted EVs that occur in vivo. Organoids provide a controlled system that allows investigators to assess changes in EV secretion and composition from cells, in response to various stimuli. Utilizing organoid cell cultures as a model to evaluate EV function in reproduction has been limited, thus far. Therefore, the goal of this paper is to review and compare organoids to other current in vitro cell culture models used to study female reproductive tissues, with an emphasis on EVs secreted or accepted by reproductive tissue-derived organoids. A literature search was performed between May and July 2021, using NCBI PubMed, including the search terms female and reproduction, and spheroid, organ-on-a-chip, and organoids or extracellular vesicles. Subsequent full text available articles were selected that covered studies that focused only on mammalian female reproduction. Finally, we will explore future directions for organoids in the rapidly developing field of EVs in female reproduction.

## 2. In Vitro Models Used to Study Reproductive Physiology

**Figure 1:**
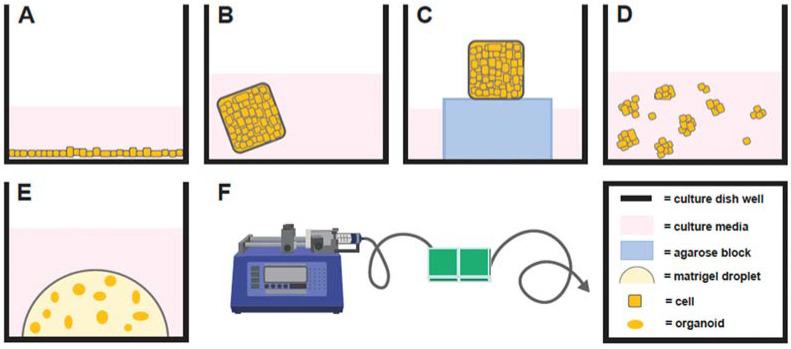
Depiction of monolayer cell culture (**A**), explant immersed in culture media (**B**), explant with a gas–fluid interface (**C**), spheroid cell culture (**D**), organoid cell culture (**E**), and organ-on-a-chip (**F**) with A–E illustrated from a lateral perspective in a single well of a multi-welled culture plate.

A variety of in vitro model systems, including monolayer, explant, spheroid, organ-on-a-chip, and organoid (Figure 1) cell cultures, have been described to study normal and abnormal female reproductive physiology. Table 1 summarizes and highlights the advantages, disadvantages, and applications of each of these in vitro culture systems.

Historically, the most widely used system to study physiology and/or disease states of female reproduction in vitro were two-dimensional (2D) monolayer culture systems (Figure 1A). Monolayers are simple, cost effective, and efficient in vitro models used to study, predominantly, one cell type. However, there are drawbacks to monolayer cell culture systems. For example, endometrial cells grown in monolayer culture undergo phenotypic changes, such as the loss of normal polarity and architecture, following long-term culture, which affects physiological intercellular interactions, and have demonstrated a loss in normal physiological response to exogenous hormones, after only a couple of passages [1,2]. In addition, primary cells in a monolayer can only be maintained for a limited time before senescence. Due to these limitations, the development of more in vivo-like cell culture models has been actively pursued for many years and has led to the development of a variety of 3D cell culture systems.

### 2.1. Explants

Several 3D cell culture systems have been described (Table 1). Explant cultures have been reported for many decades and are a 3D model system involving whole pieces of tissue placed in culture (Figure 1B). While explant cultures are simple to use, their drawbacks primarily involve a short culture time before tissue degeneration. For example, mare endometrial explants immersed in culture media degenerate significantly over a 48-h culture period [3]. An alternative equine endometrial explant model that utilized a gas–fluid interface by placing explants on agarose blocks partially immersed in culture media (Figure 1C) facilitated maintenance of tissue for 5 days in culture, but often formed a central core of necrosis [4,5]. However, long-term cultures (weeks to months) are necessary to fully recapitulate physiological reproductive processes over one or multiple estrous cycles or to evaluate pathologies with an insidious onset.

### 2.2. Spheroids

Spheroids are 3D cell clusters that do not require an extracellular matrix to form and cannot self-assemble or proliferate long term (Figure 1D) [6]. Similar to explants, spheroids often form a necrotic core, which impacts studies involving EVs, as apoptotic bodies are released by these dying cells, which can confound data because the size of apoptotic bodies overlaps with other EV types (see below) [7]. Developing spheroids with endometrial glandular tissue gave rise to a new generation of 3D cell cultures in domestic animals, which included the first canine 3D endometrial gland and stromal cell co-cultures [8]. However, this co-culture system demonstrated tissue culture for only 96 h [8,9]. A more recent report utilized feline endometrial cells, which involved greater than 75% epithelial cells, cultured with a 3% Matrigel^®^ extracellular matrix [10,11]. Under these conditions, the cells responded to estradiol and progesterone treatments with alterations in spheroid-shape patterns, by becoming ‘tube-like’ or ‘gland-like’, but no decidualization gene expression changes in response to hormone treatments were demonstrated. Furthermore, these cells were cultured short term (1–2 weeks) [10,11]. While these culture models may be more physiologically relevant than monolayer cell cultures, spheroids from reproductive tissues are still limited, as they cannot be maintained for long periods in culture before degenerating.

Another reproductive tissue that has been challenging to recapitulate with normal physiology in vitro is the placenta. Equine chorionic girdle cells, which are horse-specific specialized trophoblast cells, were cultured in a dish, either with or without 10% fetal calf serum. The chorionic girdle cells cultured with serum adhered to the culture flask as a monolayer, but the cells cultured without serum formed non-adherent spherical clumps, referred to as ‘vesicles’ by the authors [11]. These ‘vesicles’ would be referred to as ‘spheroids’ using current terminology and were cultured for approximately 7 days [12]. While this report represented a critical breakthrough by maintaining chorionic girdle in a non-explant 3D cell culture, a longer culture period with the maintenance of physiologically functioning chorionic girdle cells is necessary to evaluate early equine pregnancy effectively in vitro to allow a greater understanding of placental cellular interactions that are challenging to assess in vivo.

### 2.3. Organs-on-a-Chip

Another type of 3D cell culture system is one that uses microfluidics and is often referred to as ‘organs-on-a-chip’ (Figure 1F; for review see [13]). This model was initially described for ‘oviducts-on-a-chip’ and ‘endometrium-on-a-chip’ in women [14,15]. Organs-on-a-chip utilize microfluidics, which creates shear stress, or a force parallel to the cells, with the movement of culture media through channels in a device containing explants or cells. A pump system allows for precise changes in culture media composition, which is useful when treating cells with hormones. For example, cells could be exposed to luteinizing hormone (LH) to replicate the LH surge or to cyclical waves of follicle stimulating hormone (FSH) treatments. An advantage of this in vitro model is that the microfluidic system can be designed so that the cells of different origins can be maintained separately, while interacting, such as through the use of a semipermeable membrane. One example is perivascular stromal cells and endothelial vascular cells of women’s endometrium that required the shear stress of fluidic movement for the formation of intercellular tight junctions to successfully model the in vivo endometrium [15]. Furthermore, another group recently described an endometrium-on-a-chip model utilizing human endometrial stromal cells, epithelial cells, and vascular endothelial cells, which responds to the emergency contraceptive levonorgestrel with dose-dependent, physiological cellular apoptotic changes and blood vessel regression [16].

These organs-on-a-chip also have been reported in bovine and canine species, utilizing an ‘oviduct-on-a-chip’ model [17,18] and in bovine, utilizing an ‘endometrium-on-a-chip’ model [19]. The cow oviduct-on-a-chip was used to support in vitro fertilization (IVF) and early embryo development [17]. Unfortunately, the rate of development to 8–16 cell stages in this 3D system was reduced compared to conventional IVF. However, global methylation and transcriptome of embryos, produced in the ‘organ-on-a-chip’ system, were more similar to in vivo-produced embryos compared to embryos produced using conventional IVF [17]. These ‘organs-on-a-chip’ also can be used to study different pathologies. For example, the ‘oviduct-on-a-chip’ system described in canines used a microfluidic system, in conjunction with gene editing, to study serous tubal intraepithelial carcinoma [18] as a translational model. This model demonstrated biomimicry of this condition, with appropriate changes in cellular morphology and genetic expression. A bovine ‘endometrium-on-a-chip’ was designed to study how insulin and glucose affect endometrial function [19], and the authors found that high glucose altered gene and protein expression of the stromal and epithelial endometrial cells. Thus, ‘organ-on-a-chip’ systems can be a powerful tool to study female reproductive physiology and recapitulate the dynamic pathological changes that occur in vivo. However, a limiting factor is that these tend to be more labor-intensive and costly due to the complexity of the system.

### 2.4. Synthetic Embryology

Synthetic embryology, a method of developing embryo-like structures in vitro, can utilize 3D cell cultures to model the early developing conceptus using pluripotent stem cells (for review, see [20,21]). These 3D cultures include blastoids, embryoids, and gastruloids, depending on the developmental stage of the synthetic conceptus. Embryoids are stem cell models that resemble embryos. Blastoids are a type of embryoid that resemble blastocysts, and gastruloids resemble a gastrulating embryo. Because human embryos are difficult to obtain and culture for research purposes due to negative ethical implications, synthetic embryology provides an alternative for in vitro evaluation of direct embryo interactions with other tissue types, such as the endometrium, and their cellular communication, such as through EVs.

### 2.5. Organoids

Organoids are grown from stem cells or organ progenitor cells, which are a more differentiated type of stem cell, that self-assemble and remain both genetically and phenotypically stable throughout long-term (months) culture [22,23] (Figure 1E). The generation of organoids occurs by providing cells with a specialized culture medium, containing various supplements that allow cells to multiply and self-assemble in vitro, and enables cells to proliferate in multiple layers and return to the structure and function observed in vivo [24]. The specific mechanism that enables the genetically encoded self-assembly, which utilizes local activation of genetic patterning systems and signaling for tissue assembly, is currently unknown in organoids [25,26]; however, the mechanism appears to involve cellular exposure to specific morphogens that activate developmental signaling pathways to trigger the self-assembly [27]. The following sections describe the use of organoids from various reproductive tissues.

#### 2.5.1. Oviductal Organoids

There are a limited number of reports on female reproductive tissue-derived organoids (Table 2), and only mouse and human oviductal (fallopian tube) organoids have been described [28,29]. Oviductal organoids, derived from human oviductal cells, proliferated long term (approximately 10 months), were composed of both ciliated and secretory cells, and displayed in vivo-like changes in gene expression when exposed to estradiol and progesterone [29]. Mouse oviductal organoids also survived long term (up to 6 months), but they were derived from specific anatomical and functional sections of the oviducts: fimbria, middle oviduct, and uterotubal junction [28]. The cells from the fimbria portion of the oviduct displayed the greatest organoid-forming rate, followed by the middle section of the oviduct, and finally, a very low growth rate was reported for cells collected from the uterotubal junction. These findings indicate that progenitor cells may be more numerous toward the fimbria and less numerous toward the uterotubal junction [28]. Furthermore, a recent report found that cells derived from the proximal and distal portions of the mouse oviduct for organoid culture have distinct cell lineages by utilizing single-cell RNA sequencing (scRNA-seq) [30]. Gene and protein expression of *PAX2*, a gene associated with Müllerian duct cells of secretory function, is expressed in cells toward the uterotubal junction but not toward the infundibulum, and the *WT1* gene and protein expression, which is associated with normal cell development and survival, is expressed toward the infundibulum but not toward the uterotubal junction [30]. These findings may explain the variable organoid growth rates between the proximal and distal parts of the mouse oviduct, which was reported by both groups [28,30]. This group also demonstrated that successful oviductal organoid growth could occur with a simplified culture media, containing fewer supplements compared to the original culture media reported for human oviductal organoids, which facilitates a reduction in the financial costs associated with organoid cell culture systems [28].

Oviductal organoids also have been reported to mimic pathological conditions. One group utilized organoids developed from human-induced pluripotent stem cell (iPSC) lines, with *BRCA1* mutation, that maintained cellular abnormalities associated with serous tubal intraepithelial carcinoma for at least four months in culture [31]. Furthermore, a patient’s disease severity was correlated with the organoid phenotype [31], which supports the use of organoids for personalized medicine.

#### 2.5.2. Endometrial Organoids

Organoids derived from endometrial epithelial cells have been described in mice, women, domestic horses, and endangered equids [32,33,34,35,36]. All of these endometrial organoids can elicit physiological responses after exposure to reproductive hormones [1,32,33,34,36]. For example, equine endometrial organoids responded to oxytocin hormone treatment with changes in *PTGS2*, *PGES*, and *OXTR*, which are genes associated with the prostaglandin synthesis cascade [36]. Cleverly, one group recently reported the successful generation of endometrial organoids from menstrual secretions in women, which was determined by confirming that endometrial organoids derived from biopsies and menstrual secretions display the same transcriptome signature, efficiency, proliferation rate, and response to exogenous steroid hormones, and precludes the need for tissue biopsies [37].

Limitations of these endometrial organoid models are the lack of stromal cells and the extracellular matrix requirement, which is often of animal cell origin and has batch variability. As stromal and epithelial cells comprise the primary cell types associated with the endometrium, in vitro cell cultures are more similar to in vivo if they reflect this multi-cellular environment. To address these limitations, organoid co-culture of endometrial epithelial and stromal cells using 3D porous collagen scaffolds [38] or agarose 3D Petri Dishes [39] was recently described, which have facilitated the development of epithelial and stromal multicellular endometrial organoids in women that are more anatomically relevant. However, these non-extracellular matrix organoid systems are more challenging to maintain long term than epithelial organoids in Matrigel^®^. Culture of endometrial epithelial cell organoids on a scaffold with stromal cell seeding was reported only up to 10 days [38]. With the 3D petri dishes, cells were established for 7 days then exposed to steroid hormones for 14 days, but growth beyond this was not discussed [39]. Cellular proliferation in both of these models is much shorter than the months that organoids suspended in an extracellular matrix are maintained for.

Finally, human endometrial epithelial organoids were utilized to demonstrate that fluid produced by the apical side of the organoids into the organoid lumen is biochemically different from fluid produced by the basal side of the organoid into the surrounding conditioned media, with 17 unique metabolites in the intra-organoid fluid and 69 unique metabolites in the extra-organoid fluid [40], which indicates the regulation of particle movement across organoid cell layers. Of note, glucose was reduced in the luminal fluid of the organoids compared to the extra-organoid fluid, which is similar to women in vivo, where intrauterine fluid displays relative hypoglycemia [40]. Furthermore, oxidized nicotinamide adenine dinucleotide (NAD^+^) also was elevated in the intra-organoid fluid compared to the extra-organoid fluid, which was demonstrated to be necessary in mice during pregnancy, to prevent congenital malformations and miscarriages [40,41]. Initially, this group had to utilize micromanipulation to access the intra-organoid fluid but later developed a high-throughput method, using centrifugation, to disrupt the organoids to allow intra-organoid fluid release without impairing cellular viability [40].

Several studies have utilized organoids derived from human endometrium as model systems for pathologies and to evaluate endometrial receptivity. One study developed organoids utilizing endometrium collected from healthy women and those with adenomyosis to study infertility and determined that organoids generated using the pathological tissues maintained their diseased phenotype [42]. Another group generated organoids from women with healthy endometrium and those with endometriosis, to assess epigenetic methylation of *HOX* genes and their cofactors, and found that organoids developed with endometriosis tissues maintained the epigenetic patterns for the majority of the evaluated sites [43]. Thus, endometrial organoids may be an appropriate in vitro model to study endometriosis in women. Furthermore, another group produced organoids utilizing tissues with several types of pathological conditions, including endometriosis, neoplastic subtypes, endometrial hyperplasia, and Lynch syndrome, which maintain their associated genome and transcriptome [44]. A further application was described using endometrial organoids as a model for embryo implantation [45]. This group demonstrated that organoids respond to hormonal treatments to mimic molecular and morphological changes, associated with the proliferative and secretory phases of the menstrual cycle and that glycodelin-A (GdA), which is associated with endometrial receptivity, varies between organoids developed from healthy endometrium and tissue with endometriosis [45]. The same group expanded upon this study by co-culturing endometrial organoids with conditioned medium collected from growing embryo cultures and found that GdA increased in the treated organoids compared to the controls [46]. This data then was applied during an in vivo trial and found that clinical pregnancy rates increased when the conditioned medium, collected from growing embryos, was included during embryo transfer [46]. All of these studies indicate that endometrial organoids are a relevant in vitro model to evaluate various pathologies and normal endometrial physiology, which may result in disease prevention and/or treatment and improve pregnancy rates.

#### 2.5.3. Trophoblast Organoids

Placental cell (i.e., trophoblast) organoids have been reported as a model to study human placentation [47,48]. First trimester (6–9 weeks of gestation) placental fragments were embedded in the extracellular matrix Matrigel^®^ and overlaid with culture media, containing various supplements [47,48]. Organoids derived from trophoblast cells demonstrated differentiation, into both syncytiotrophoblast and extravillous trophoblast cells, and were cultured long term (over a year), remaining genetically stable throughout this culture period. Extravillous trophoblast organoids expressed HLA-G, which is expressed in extravillous trophoblast cells in vivo, and do not express ITGA2, which is associated with cytotrophoblast progenitor cells and is present in the syncytiotrophoblast organoids [47,49,50]. These organoids also remained functionally active, as evidenced by the ability of the syncytiotrophoblasts to secrete placenta-specific peptides, such as the maternal recognition of pregnancy hormone human chorionic gonadotropin (hCG), which was detected by ELISA and a digital home pregnancy test.

#### 2.5.4. Ovarian Organoids

Several groups have recently reported the culture of organoids derived from various ovarian neoplasia subtypes [51,52,53,54]. Organoids generated from individual patient tumors were characterized morphologically and genetically, and trialed with various therapeutic agents, to determine the best treatment to facilitate tumor regression in a particular patient [51,52]. These groups demonstrated how ovarian cancer can be studied in greater detail in vitro, with the use of a 3D organoid model as a diagnostic device. Research using organoids derived from individual patient cells may provide a novel method for personalized medicine, with targeted therapeutics for more successful treatment.

#### 2.5.5. Organoid Applications for Animal Reproduction

Organoids provide a physiologically-representative, less labor-intensive in vitro model, for the study of reproductive physiology, pathology, and therapeutics. Future studies utilizing a 3D organoid system may involve the improvement of in vitro maturation of oocytes in many species, using co-culture with oviductal organoids, or in vitro culture of embryos, utilizing co-culture with oviductal and/or endometrial organoids. This may be particularly applicable for the currently unconquered canine in vitro maturation system [55] or unaccomplished equine in vitro fertilization [56]. Furthermore, normal and pathological reproductive conditions could be more effectively evaluated and potential therapeutics trialed using reproductive organoid models. For example, endometritis, which is inflammation of the uterine lining, and cystic endometrial hyperplasia (CEH)-pyometra complex in bitches are challenging to investigate in vivo due to the significant welfare concerns associated with experimental induction of these life-threatening pathologies. Developing a canine endometrial organoid model for these pathologies would facilitate the study of pathophysiology and potential novel therapeutics for these diseases.

This in vitro culture model also can be used effectively to study complex intercellular communication interactions and pathways, particularly the role of EVs in normal and pathological reproductive organ systems. Having a functional, long-term in vitro model that better recapitulates in vivo conditions than traditional in vitro models offers significant advantages when studying normal and pathological female reproductive processes by (i) reducing time requirements by performing evaluations concurrently rather than consecutively, (ii) lessening financial cost due to the high expenses of utilizing research animals, and (iii) improving research animal welfare.

**Table 2:** Advantages and disadvantages of organoids derived from the female reproductive tract in humans, mice, and horses.

Tissue Type	Species	References	Advantages	Disadvantages
Ovary	Human	[51]	Long-term growth (months); used to model ovarian cancer	Utilized Cultrex^®^ extracellular matrix, which is similar to Matrigel^®^, and can vary by batch and is of animal cell (Engelbreth-Holm-Swarm mouse sarcoma) origin
		[52,54,57]	Long-term growth (months); used to model ovarian cancer such as high grade serous ovarian cancer	Utilized Matrigel^®^ extracellular matrix which can vary by batch and is of animal cell origin
	Mouse	[58]	Long-term growth (months); used to model ovarian cancer	Utilized Matrigel^®^ (see disadvantages above)
Oviduct	Human	[29,57,59]	Long-term growth (months); used to model ovarian cancer ([57] only)	Utilized Matrigel^®^ (see disadvantages above)
		[51]	Long-term growth (months); used to model ovarian cancer	Utilized Cultrex^®^ (see disadvantages above)
	Mouse	[28,58]	Long-term growth (months); used to model ovarian cancer ([58] only)	Utilized Matrigel^®^ (see disadvantages above)
Endometrium	Human	[32,33,34]	Long-term growth (months); no specialized equipment or scaffolding required	Contained only epithelial cells (secretory and ciliated); utilized Matrigel^®^ (see disadvantages above)
		[38]	Contained both epithelial and stromal cells	Stromal cells were initially cultured in monolayers, and epithelial cells were cultured as organoids embedded in Matrigel^®^ before being seeded on a scaffold, but the culture time on the scaffold with both cell types was shorter than expected for organoid culture (10 days); required specialized scaffolding
		[39]	Contained both epithelial and stromal cells; did not require Matrigel^®^ and instead used 3D Petri Dishes^®^	Culture period was not as long-term (up to 21 days) as expected for organoid culture
	Mouse	[32]	Long-term growth (months); no specialized equipment or scaffolding required	Contained only epithelial cells; utilized Matrigel^®^ (see disadvantages above)
	Horse	[35,36]	Long-term growth; no specialized equipment or scaffolding required	Contained only epithelial cells; utilized Matrigel^®^ (see disadvantages above)
Trophoblast	Human	[47]	Long-term growth (months); no specialized equipment or scaffolding required	Utilized Matrigel^®^ (see disadvantages above)
Cervix	Human	[60,61]	Long-term growth (months); no specialized equipment or scaffolding required	Contained only epithelial cells; utilized Matrigel^®^ (see disadvantages above)
	Mouse	[60]	Long-term growth (months); no specialized equipment or scaffolding required	Contained only epithelial cells; utilized Matrigel^®^ (see disadvantages above)
Vagina	Mouse	[62]	Long-term growth (months); no specialized equipment or scaffolding required	Contained only epithelial cells; utilized Matrigel^®^ (see disadvantages above)

## 3. Extracellular Vesicles (EVs)

Intercellular communication in reproductive tissues involves many processes, including direct cellular interaction through gap junctions, autocrine and paracrine signaling, endocrine communication with hormones, and communication through EVs. The standard guidelines for EV terminology, physiology, separation, and characterization are an important component for understanding the biogenesis of organoid-derived EVs and their potential research and therapeutic roles. EVs are nanoparticles that contain bioactive molecules, such as nucleic acids and proteins, and are surrounded by a lipid bilayer. They cannot replicate (no functional nucleus) and are naturally secreted by cells, and their primary function is intercellular communication [63]. Historically, EVs have been characterized by their biogenesis pathway and size, into three categories: (i) microvesicles, (ii) exosomes, and (iii) apoptotic bodies. Microvesicles range from 100–1000 nm in diameter and are formed by the outward budding of the plasma membrane [64]. Exosomes have a diameter of approximately 30–150 nm and are formed by the inward budding of the outer membrane of intracellular multivesicular bodies [64]. Degenerating cells release apoptotic bodies that are ~50–5000 nm in diameter and contain intact organelles, chromatin, and glycosylated proteins [64]. For a full review of EVs and current standards for EV publications, see [63,65,66,67].

### EVs in Reproduction

EVs play a pivotal role in female reproduction (for review, see [68]) and are secreted by cells associated with the ovary, oviduct, uterus, and conceptus. In recent publications, EVs have been described in the follicular fluid of women, mares, cows, sows, and queens and have a significant impact on oocyte maturation, development, metabolism, and protection against heat stress [69,70,71,72,73,74,75]. Specifically, the miRNA profile of EVs in follicular fluid in women is distinct in oocytes that further develop into high-quality embryos, compared to those that are not high quality [69]. Bovine follicular fluid EVs are involved in modulating the arrest of oocyte meiosis [71], and they are involved in heat stress modulation in cows [70,74]. Furthermore, feline oocytes vitrified in the presence of follicular fluid EVs impacted the ability of the frozen–thawed oocytes to resume meiosis [73]. EVs isolated from oviductal fluid impact sperm viability, sperm motility, formation of the sperm reservoir, oocyte maturation, sperm–oocyte binding, fertilization, and embryo development and quality [76,77,78,79,80,81,82,83]. Furthermore, EVs isolated from uterine fluid in cows were supplemented in in vitro culture medium, which resulted in improved somatic cell nuclear transfer (SCNT) embryonic development and blastocyst quality [84]. Lastly, vaginal fluid collected from mice during hormonally induced estrus contained EVs, which, when co-incubated with sperm, played a role in preventing premature capacitation and the acrosome reaction [85]. These studies indicate that in vivo-produced EVs impact reproduction, but a sustainable source of EVs, such as those produced in vitro, are required for implementation in breeding programs.

Utilizing in vitro models, the role of EVs from specific cells and/or tissue types can be evaluated more effectively than in vivo and in a more sustainable manner. EVs secreted by bovine amniotic cell monolayer cultures improved the hatching percentage and pregnancy rates of cryopreserved in vitro-produced embryos, compared to cryopreserved embryos not treated with amniotic EVs, by modulating the expression of specific miRNAs [86]. The same group also reported the use of amniotic EVs as a regenerative medicine therapy for chronic endometritis in a mare [87]. While additional research must be performed to validate this case report (n = 1), regenerative treatments for the management of the difficult condition of chronic equine endometritis is an exciting prospect, and this technology could be translated to the treatment of endometritis in women and other mammalian species.

Co-culture of EVs secreted by estrual oviductal spheroids with canine oocytes improved in vitro maturation to metaphase II [82], which occurs in the oviduct of bitches and is still a significant challenge to achieve in vitro. Furthermore, EVs secreted into oviductal fluid in vivo are beneficial after co-culture with sperm, by impacting motility, viability, and acrosome integrity [79,81,88,89]. The oviductal fluid utilized in these studies required flushing oviducts ex vivo, which were collected either post-mortem or post-ovariohysterectomy, as a source of EVs, which is not a commercially viable option and impossible in some non-domestic species. However, oviductal organoids may be an alternative source of EVs that are more similar to EVs derived from oviductal tissues in vivo than those produced by 2D cell cultures.

## 4. Current Reports of EVs with Organoid Cell Culture Models

While there are many reports on the study of EVs in vitro, there are few reports on the presence, role, or function of EVs derived from cells in organoid models, especially organoids derived from reproductive tissues. In vitro models are necessary for the evaluation of EV content and/or function because the role of EVs, produced by specific cells or tissue types under specific conditions, cannot be studied in vivo, effectively, without confounding factors. For example, EVs secreted by mural granulosa cells in ovarian follicles can be best studied in vitro, without EV contamination from cumulus cells.

**Figure 2:**
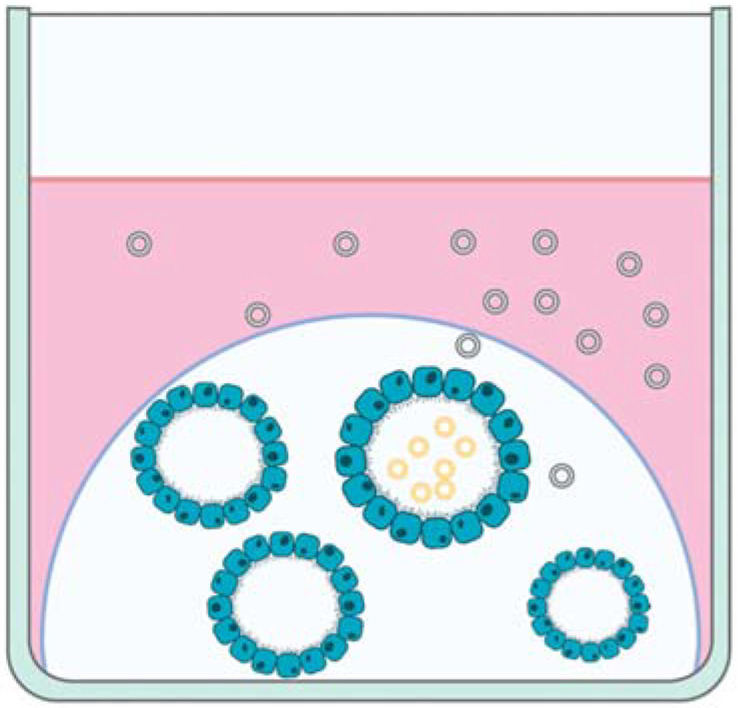
Depiction of extracellular vesicles (EVs) secreted by the apical (yellow EVs) and basal (green EVs) aspects of organoids in cell culture.

Most research evaluating EVs secreted by organoids has focused on cancer, cardiac repair, and stem cells [7], but the data from these studies may be extrapolated for use in reproductive organoids and EVs. For example, while evaluating stem cells of the oral cavity, the authors compared 2D and 3D cell culture models and concluded that 3D cell cultures secrete a significantly higher amount of EVs [90]. Therefore, extrapolation of this data may indicate that 3D reproductive cell cultures, such as reproductive organoids, may be likely to produce more EVs than 2D reproductive monolayer cultures. Furthermore, similar to biochemical differences in secretions from the apical and basal cellular surfaces of endometrial organoids [40], colon carcinoma organoids secreted EVs from the apical surface that are a distinct population with a differing proteomic profile compared to EVs secreted by the basolateral surface of the organoids [91] (Figure 2). These data indicate that EVs secreted from the apical and basolateral surfaces of reproductive organoids should also be compared to each other to evaluate differences between the EV populations and to determine which population is more similar to EVs produced by that tissue in vivo.

Several groups have evaluated EVs secreted by neoplastic cells to understand the pathophysiology of neoplastic conditions to develop novel diagnostic tools. Co-incubation of EVs secreted by esophageal adenocarcinoma with a gastric organoid model resulted in the induction of a neoplastic phenotype, which revealed specific neoplastic-inducing miRNAs in the EV cargo [92], which was significant as this demonstrated how neoplastic cells may utilize EVs for tumor growth and metastasis. Another group collected serum from human patients with colorectal adenoma to isolate EVs and tissue, to establish organoids to produce EVs, which were utilized to screen EVs for miRNAs associated with this cancer to determine if a ‘liquid biopsy’ could be an alternative screening process to stool sample collection for colorectal cancer [93].

Intestinal organoids are another field that has demonstrated the wide uses for this model system. Intestinal organoids have been used to demonstrate that EVs play a role in inflammatory immune modulation and that opioids impair immune modulation by the secreted EVs [94]. Another report describes how treating intestinal stem cell niche organoids with EVs secreted by intestinal stromal cells transmits Wnt and epidermal growth factor (EGF) activities, which are necessary to maintain the ‘stemness’ of these intestinal stem cell organoids [95]. In the field of parasitology, cecal organoids were treated with EVs from whipworms (*Trichuris muris*) to evaluate host–parasite interactions [96]. These findings demonstrate the diverse applications that can be useful when studying reproductive organoids. These may include EVs secreted from reproductive organoids, the interaction of EVs secreted by other tissues with reproductive organoids, and trialing EVs as diagnostics and therapeutics with reproductive organoids.

**Figure 3:**
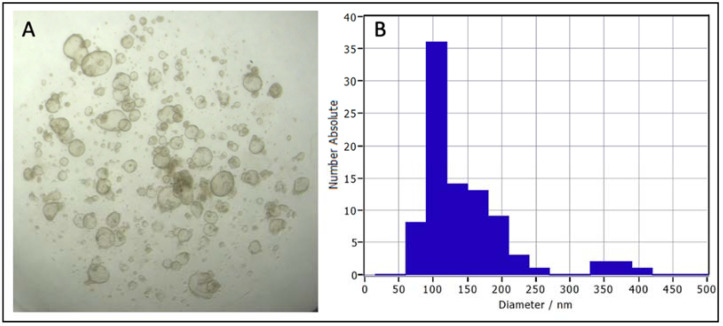
Bovine oviductal organoids after 14 days in culture (**A**), and a graph of nanoparticle tracking analysis (NTA) using ZetaView^®^ technology of the size distribution of extracellular vesicles collected from bovine oviductal organoid conditioned media following differential ultracentrifugation (**B**).

Evaluating EVs from a specific cell type is not possible in vivo, but a relevant, physiological, in vitro 3D model can facilitate this. EVs derived from bovine oviductal fluid (in vivo) versus bovine oviductal epithelial cells, cultured in a 2D monolayer (in vitro), revealed differences between the protein content of these two EV sources [76], which is a significant limitation of 2D culture models. These limitations included a lack of OVGP1 (MUC9) in EVs produced by the monolayers but its presence in in vivo-derived oviductal EVs, which is critical for early embryo development [97]. As organoids are more similar to in vivo architecture and physiology than 2D monolayer cultures, organoids may be a more relevant source for evaluating EV function and as an alternative source of EVs to improve artificial reproductive technologies. To support this, 3D spheroid culture of cervical cancer cells demonstrated that the nucleic acid cargo of EVs secreted by 3D culture is more similar to in vivo-derived EVs than corresponding 2D monolayers [97,98]. Although research evaluating EVs secreted by organoids is emerging, to the authors’ knowledge, no data on EVs secreted by reproductive organoids has been published. Early data from our laboratory demonstrate the release of EVs by bovine oviductal organoids (unpublished data; Figure 3). These bovine oviductal organoids were established and maintained in the same manner as described for equine endometrial organoids [36], and the conditioned media collected from these organoids was evaluated by nanoparticle tracking analysis (NTA), using ZetaView^®^ technology. While these preliminary data demonstrate that reproductive tissue organoids secrete EVs, a large gap in our knowledge exists regarding the cargo and function of these EVs and the utility to study these in organoid systems of female reproductive physiology.

## 5. Future Directions for Reproductive Organoids and EVs

### 5.1. Increased Understanding of Physiology Using Organoids and EVs

Tissue interactions may be more effectively evaluated in vitro than in vivo utilizing EVs. Placental EVs could be added to endometrial organoid cell cultures to determine the signaling role between the conceptus and the uterine environment. Furthermore, placental EVs in women can be found in the maternal plasma [99], which indicates that these placental EVs interact with non-reproductive organ systems. Determining the exact role of placentally derived EVs on non-reproductive organ systems may be accomplished best in vitro, by isolating placental EVs and co-culturing with organoids for their direct physiological impact on the target tissue, such as placental EV interaction with hypothalamic and/or pituitary organoids. These types of ‘long-distance’ tissue interactions may be most effectively evaluated using EVs and organoid culture systems.

Another area to explore is the expected EV production per organoid. The EVs produced per organoid are likely dependent on the cell origin and total cells per organoid, which might be determined by extrapolating from the organoid diameter. This is important because EV production per organoid could have significant implications for the commercial production of EVs for therapeutics.

### 5.2. Increasing EV Production by Organoids

Production and separation of EVs secreted by organoids can be time-intensive to generate a large number of EVs, which would be required for commercial production for therapeutic use. Alternative models for high-throughput production of EVs are needed. Bioreactors may be a culture method that can increase the total number of organoids and EVs produced and reduce labor input, to facilitate the commercial application of organoid/EV biotechnology. Bioreactors have been demonstrated to improve the functionality of organoids [100], and another group fabricated miniature spinning bioreactors with a 3D printer and commercial hardware to increase throughput of brain organoids [101]. By utilizing some of these technologies, organoids derived from reproductive cells and their EVs may be generated more efficiently to (i) facilitate a more powerful tool to study reproductive pathology, physiology, and fertility, (ii) develop an important source of EVs for use in regenerative medicine, and (iii) improve the efficacy and efficiency of artificial reproductive technologies.

Additional automation technologies could also increase organoid throughput. Several commercial companies have reported 3D bioprinters for automating the extracellular matrix plating process, which traditionally relies on the speed of the technician to maintain temperature control of the extracellular matrix, and accuracy of the technician to distribute extracellular matrix droplets in the appropriate orientation in the culture dish. Automation increases the speed, accuracy and, ultimately, performance of these extracellular matrices to increase organoid and EV production.

### 5.3. EVs Produced by Organoids as Therapeutics

As in vivo-produced EVs from the reproductive tract improve various sperm parameters, oocyte maturation, and pregnancy rates after the transfer of EV-treated embryos, an alternative in vitro source of EVs from a physiologically relevant model, such as organoids, would facilitate the sustainable and commercial use of EVs for artificial reproductive technologies. Production of EVs in vitro can be scaled to meet clinical in vivo needs, while only a finite amount of EVs can be collected from in vivo sources. Additional research will be required to determine whether large-scale, commercial production of EVs is ideal or if individualized EV production is a more appropriate objective. One concern that must be evaluated prior to the use of in vitro-produced EVs as an in vivo therapeutic is the potential epigenetic alteration that may occur as a result of these EV treatments. A recent publication, using a mouse model with some supporting human data, demonstrated that EVs produced by stressed epididymal cells deliver miRNA to sperm that, ultimately, result in offspring that display a physiological stress phenotype [102]. These long-term implications for offspring must be more fully evaluated to ensure that in vitro-produced EVs, used for artificial reproductive technologies, do not result in undesirable epigenetic alteration of the offspring. Additional studies may include establishing favorable conditions in vitro that result in a desirable offspring phenotype.

Organoids also may be utilized to treat reproductive pathologies or to promote the likelihood of pregnancy. Similar to how a tissue’s microbiome must be maintained for healthy organ function, perhaps a healthy tissue secretes a specific population of beneficial EVs. If this is true, perhaps EVs from healthy reproductive tissue, such as from the uterus, may be supplemented to tissues/organs that have undergone pathological changes associated with disease or infection, such as endometritis. Furthermore, EVs may be involved in maternal recognition of pregnancy (MRP) signals [68,103]. In females with early embryonic loss, EVs containing these MRP signals could be supplemented to improve pregnancy rates. Once these MRP molecular signals are fully elucidated, organoids could be established to increase the production of these critical EVs to improve the maintenance of pregnancy.

### 5.4. Organoids and Their EVs as Biomarkers

The role of organoids in personalized medicine is a rapidly emerging area of research. Tissue from a specific patient could be grown in an organoid culture system and various therapeutics could be studied in tandem to determine the most appropriate treatment for that patient’s pathology. This would be similar to bacterial culture from the endometrium of an individual with endometritis, followed by sensitivity trials with a range of antibiotics, to determine the optimum therapeutic. Another case for individualized medicine would be the collection and evaluation of EVs secreted by organoids derived from a pathology to determine appropriate biomarkers in a liquid biopsy for a specific pathology. To accomplish this, a fluid sample, such as blood or urine, would be collected, the EVs in the sample evaluated, and a pathology associated with certain EV cargo or surface molecules may be diagnosed in lieu of an invasive tissue biopsy.

The hemochorial placenta in humans is the most invasive type of placentation and allows proteins, such as those associated with immunity, to cross readily from the mother into the fetal blood. Domestic animals have less invasion of the placenta into the maternal endometrial cell layers, such as endotheliochorial, synepitheliochorial, and epitheliochorial placentation, which allows very little protein to cross between the dam and fetus. However, as EVs are lipid-bound nanoparticles, they likely have a greater ability to cross between the fetus and dam in domestic animals. How much access EVs have to cross between the placental layers of the dam and fetus for each non-human placental type has yet to be evaluated. Depending on the amount of EV crossover, EVs in domestic animal maternal blood may be a biomarker for fetal or placental health [68]. These EV biomarkers may be initially studied using an organoid system to determine the cargo of EVs secreted by tissue derived from a normal and inflamed placenta (placentitis).

In addition, gestational aging may be possible by measurement of blood EV concentration [68], depending on the level of EV crossover from the fetus to the dam in each domestic species. EV concentration in the maternal blood plasma of pregnant women increases as gestation advances. EVs are 50-fold higher in pregnant than in non-pregnant women, and EV concentration increases with each subsequent gestational trimester [99]. While these maternal blood plasma EVs from pregnant women were characterized using western blot for surface proteins, transmission electron microscopy, and nanoparticle tracking analysis (NTA) for size and concentration, future studies, such as those in domestic animals, could include determining whether the EV cargo changes throughout gestation. If a pattern of fetal EV concentration and/or cargo could be mapped for each domestic species, EVs could be a minimally invasive method for pregnancy diagnosis and gestational aging, which may reduce labor and financial cost associated with pregnancy diagnosis and gestational aging in domestic animals. Furthermore, production efficiency in livestock industries, such as the dairy and beef industries, would be improved by this potential gestational aging and/or fetal health biomarker, and canine health may be improved by providing additional supportive evidence for gestational aging in pregnant bitches to ensure appropriate cesarian section timing that will prevent premature pups and neonatal loss. This initial biomarker mapping may be best accomplished using an in vitro organoid model system, in conjunction with in vivo sampling.

## 6. Conclusions

3D in vitro cell culture models have been utilized for many years and have evolved to more closely mimic in vivo reproductive anatomy and physiology. Recently, new applications of cell culture technologies, such as utilizing extracellular matrices in a 3D droplet, in conjunction with specific culture media to allow organoid generation, have facilitated the applicability of these 3D models to study reproductive physiology and pathophysiology. Development of organoids from reproductive tissues in humans and domestic and wildlife species has been limited, despite the financial, welfare, and time benefits they offer over in vivo models. In particular, 3D cell culture models in wildlife species have greatly improved the safety of both animals and their human caretakers. Utilizing organoids for the study of specific EV functions is promising in the field of reproduction. EVs produced by female reproductive organoids can be used diagnostically, therapeutically, and to improve understanding of normal and pathological physiology of reproduction in many species.

## Figures and Tables

**Table 1:** Comparison of different in vitro models.

Cell Culture Model	Advantages	Disadvantages	Applications
Monolayer	Minimal supplies required; easy to establish and maintain	Lose normal function quickly through differentiation or senescence; a single cell layer is not always physiologically relevant	Experiments to evaluate basic cell function; can be used for basic drug screening tests but not ideal for evaluating penetration of drugs in a 3D environment
Explant	Minimal supplies required; easy to establish; 3D structure; contains all cell types and orientation of in vivo environment	Short culture time (<1 week) while maintaining cell viability; cell necrosis in tissue center	Short-term culture experiments with the benefit of containing all cell types and structure present in vivo
Spheroid	Minimal supplies required; easy to establish; 3D structure is more similar to in vivo environment	Cannot self-assemble; no long-term proliferation; cell necrosis in spheroid center	Typically used for 3D cell culture using cell lines; improvement over monolayer cell culture in cellular orientation but not as similar to in vivo orientation and function as organoids
Organ-on-a-chip	Fluidics provides a source of continually flowing culture media that mimics the vascular system by delivering nutrients and removing waste products	Higher financial and time cost associated with fabrication and maintenance of the culture system; often cells are maintained in monolayers within the microfluidic environment which is not representative of the in vivo environment; requires some engineering expertise	Cell culture experiments with a precise hormone delivery schedule; has been demonstrated for use in the evaluation of multiple organ interactions by linking various organs-on-a-chip
Organoid	Self-assembly; maintains cellular polarity; long-term proliferation (months); maintains in vivo phenotype, genotype, and function	Typically a static model which does not allow for constant delivery of fresh nutrients and removal of waste; can be time-intensive if growing many organoids; often requires the use of Matrigel^®^ which has batch variability and is of animal cell origin	Ideal for evaluating cellular function and drug trials in a manner most similar to the in vivo environment; less ideal if direct contact is needed for cellular interaction (e.g., sperm binding to oviductal cells) due to the traditional extracellular matrix requirement (Matrigel^®^)

## Data Availability

Data sharing not applicable.
